# Imprinted CDKN1C Is a Tumor Suppressor in Rhabdoid Tumor and Activated by Restoration of SMARCB1 and Histone Deacetylase Inhibitors

**DOI:** 10.1371/journal.pone.0004482

**Published:** 2009-02-16

**Authors:** Elizabeth M. Algar, Andrea Muscat, Vinod Dagar, Christian Rickert, C. W. Chow, Jaclyn A. Biegel, Paul G. Ekert, Richard Saffery, Jeff Craig, Ricky W. Johnstone, David M. Ashley

**Affiliations:** 1 Children's Cancer Centre research laboratory, Murdoch Children's Research Institute, Royal Children's Hospital, Parkville, Australia; 2 Department of Paediatrics University of Melbourne, Royal Children's Hospital, Parkville, Australia; 3 Anatomical Pathology, Royal Children's Hospital, Parkville, Melbourne, Australia; 4 Departments of Pediatrics and Pathology Children's Hospital of Philadelphia, and the University of Pennsylvania School of Medicine, Philadelphia, Pennsylvania, United States of America; 5 Epigenetics laboratory, Murdoch Children's Research Institute, Royal Children's Hospital, Parkville, Australia; 6 Peter MacCallum Cancer Centre, St Andrews Place, East Melbourne, Australia; Ordway Research Institute, United States of America

## Abstract

*SMARCB1* is deleted in rhabdoid tumor, an aggressive paediatric malignancy affecting the kidney and CNS. We hypothesized that the oncogenic pathway in rhabdoid tumors involved epigenetic silencing of key cell cycle regulators as a consequence of altered chromatin-remodelling, attributable to loss of *SMARCB1*, and that this hypothesis if proven could provide a biological rationale for testing epigenetic therapies in this disease. We used an inducible expression system to show that the imprinted cell cycle inhibitor *CDKN1C* is a downstream target for SMARCB1 and is transcriptionally activated by increased histone H3 and H4 acetylation at the promoter. We also show that CDKN1C expression induces cell cycle arrest, CDKN1C knockdown with siRNA is associated with increased proliferation, and is able to compete against the anti-proliferative effect of restored SMARCB1 expression. The histone deacetylase inhibitor (HDACi), Romidepsin, specifically restored *CDKN1C* expression in rhabdoid tumor cells through promoter histone H3 and H4 acetylation, recapitulating the effect of SMARCB1 on *CDKNIC* allelic expression, and induced cell cycle arrest in G401 and STM91-01 rhabdoid tumor cell lines. *CDKN1C* expression was also shown to be generally absent in clinical specimens of rhabdoid tumor, however CDKN1A and CDKN1B expression persisted. Our observations suggest that maintenance of CDKN1C expression plays a critical role in preventing rhabdoid tumor growth. Significantly, we report for the first time, parallels between the molecular pathways of SMARCB1 restoration and Romidepsin treatment, and demonstrate a biological basis for the further exploration of histone deacetylase inhibitors as relevant therapeutic reagents in the treatment of rhabdoid tumor.

## Introduction

Rhabdoid tumor (RT) is an aggressive although rare tumor of infancy and early childhood resistant to conventional chemotherapies and radiotherapy. The majority of afflicted children succumb to their disease within several months of diagnosis. Rhabdoid tumors mainly arise in the kidney where they are known as rhabdoid tumours and in the central nervous system where they are referred to as Atypical Teratoid Rhabdoid Tumor (AT/RT). They are characterized genetically by deletion or allelic loss of chromosome 22q, and associated inactivating mutations or deletion of the tumor suppressor gene *SMARCB1* (OMIM 601607) [1], [2], [3], [4], [5], [6].

Homozygous deletion of *smarcb1* in mice is embryonic lethal, however, heterozygous mice develop tumors that are histologically similar to their human counterparts [7], [8], [9]. Tumor formation in mice is accelerated by coincident *p53* mutation[10] and it has been recently proposed that tumor formation associated with loss of SMARCB1 may arise due to permissive defects in cellular DNA damage response pathways [11]. Although *SMARCB1* deletion is predominantly associated with RT, recently *SMARCB1* inactivation and mutation has been described in epitheloid sarcoma and familial schwannomatosis [12], [13].

One suggested mechanism by which loss of *SMARCB1* facilitates oncogenesis is through defective cell cycle regulation. Re-expression of *SMARCB1* in human rhabdoid tumor cell lines causes G0/G1 arrest showing that restoration of *SMARCB1* expression is sufficient to suppress proliferation [14], [15]. This is associated with activation of *p16INK4a* and *CDKN1A* and down regulation of E2F target genes including *cyclin A*, *E2F1* and *CDC6*. SMARCB1 arrest is critically dependent on the presence of functional *pRB* [16]. SMARCB1 is not able to arrest cells lacking functional *pRB* and arrest can also be reversed by disruption of pRB repressor complexes through restoration of cyclin D1 and cyclin E expression. Further, constituitively active pRB1 can induce arrest in RT cell lines lacking SMARCB1.

SMARCB1 is part of an ATP dependent multiprotein SWI/SNF chromatin remodelling complex [17]. It associates with ATPase subunits Brg1 (for Brahma-related gene 1, or SNF2β and Brm (for Brahma or SNF2α). In contrast to SMARCB1, Brg1 and Brm are required for cell cycle arrest mediated by pRB. Versteege et al [16] hypothesize that Brg1 and Brm are necessary for the chromatin remodelling associated with pRB repression of E2F and that SMARCB1 has a promoting but not a primary role in this remodelling. Deletion of *Brg1* and *Brm* occurs in many cancer cell lines and is associated with gene specific changes in promoter methylation at *CD44* and *E-Cadherin* leading to hyper-methylation and gene silencing [18]. Brg1 and Brm associate directly with the promoters of these genes and a more widespread role in epigenetic regulation of gene expression during tumor progression has been proposed. The direct role of SMARCB1 in chromatin remodelling has not been extensively explored. Pan et al [19] have shown that SMARCB1 represses the *c-Fos* promoter via histone deacetylation in 293T cells and that this occurs via direct interactions between HDAC4 and SMARCB1, and Zhang et al [20] showed that interactions between HDAC1 and hSNF5/INI1 (SMARCB1) were required to repress Cyclin D.

We hypothesized that the oncogenic pathway induced by *SMARCB1* inactivation in RT may involve epigenetic silencing of key cell cycle target genes. This premise, if established, may reveal opportunities for treatment of RT with epigenetic therapies that restore the expression of key growth-regulating genes. In this work we demonstrate that the imprinted cell cycle inhibitor *CDKN1C* (OMIM 600856) is a downstream target for *SMARCB1* epigenetic regulation. SMARCB1 consistently activated CDKN1C expression via histone H3 and H4 acetylation at the *CDKN1C* promoter and the histone deacetylase inhibitor (HDACi), romidepsin, restored imprinted CDKN1C expression in RT cells through promoter histone H3 and H4 acetylation. Significantly, CDKN1C expression was absent or negligible in clinical specimens, enforced expression of CDKN1C in G401 RT cells induced cell cycle arrest and knockdown of endogenous CDKN1C increased proliferation in G401 RT cells as well as attenuating the effects of SMARCB1 re-expression on cell proliferation. Our findings show that *CDKN1C* silencing is common in RT, suggest that CDKN1C is an important regulator of RT cell growth, and that RT cell proliferation is mediated in part via suppression of CDKN1C expression attributable to deletion of SMARCB1.

## Results

### CDKN1C is up-regulated in RT cells with reconstituted expression of SMARCB1

CDKN1C (p57^Kip2^) is commonly epigenetically silenced in cancer and is the only imprinted member of the CIP KIP family of cell cycle inhibitors. We hypothesized therefore that CDKN1C (p57^Kip2^) may be a potential downstream target for SMARCB1. To examine this we expressed *SMARCB1* under the control of an inducible promoter in two renal RT cell lines, G401 and STM91-01. G401 is an established renal RT cell line homozygously deleted for *SMARCB1* [1]. STM91-01 is derived from a lung metastasis with 22q loss of heterozygosity and deletion of *SMARCB1* exons 1 to 5 on the retained allele. Deletion of SMARCB1 protein in all RT cell lines used in the study was confirmed by Western blotting and further evidence for *SMARCB1* deletion or rearrangement was obtained by southern blotting (results not shown).

**Figure 1:**
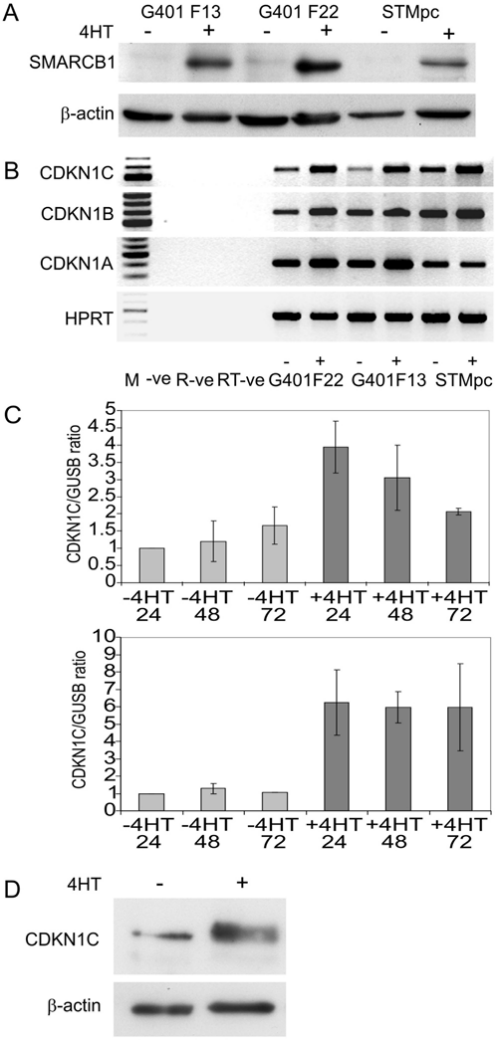
SMARCB1 induces CDKN1C expression in rhabdoid tumor cell lines. (A). Western blot showing induction of SMARCB1 protein in G401 clones F13 and F22, and in a transduced pool of STM91-01 cells, STMpc. Lanes show protein in un-induced (−) cells and in cells induced with 4HT (+). (B) Gene expression examined by RT-PCR for CDKN1C, CDKN1B, CDKN1A and the HPRT control gene in un-induced (−) and in 4HT-induced (+) cells. The -ve lane represents the PCR negative control, the RNA –ve control lane represents the negative control for reverse transcription and the RT-ve control represents a control for genomic contamination derived from the induced F22 sample. CDKN1C was amplified for 40 cycles and CDKN1B and CDKN1A for 28 cycles. All primers were located in separate exons. (C) CDKN1C expression normalized to the GUSB control gene, derived by real-time quantitative pcr in un-induced (−4HT) and in induced (+4HT) cultures of F22 cells (upper panel) and in cultures of STMpc cells (lower panel). The data represent the mean of three independent experiments and the error bars represent the standard error of the mean. (D) Western blot showing expression of endogenous CDKN1C protein pre and post SMARCB1 induction with 4HT. Immunoblotting was performed with the p57 Kip2 antibody from Cell Signaling Technologies with an exposure time of 2 minutes.

G401 clones expressing SMARCB1 under the control of a tamoxifen inducible promoter system (pcDNA3 UAS SMARCB1 and pEF puro/GEV) were selected in geneticin and puromycin. Two G401 clones, F13 and F22, showing inducible SMARCB1 expression, were selected for further investigation. A pool of STM91-01 cells (STMpc), transduced with a lentiviral vector system (pF 5×UAS Sv40 puro/ini1 and pF GEV16) was also examined. SMARCB1 protein was readily detectable 24 hours after exposure to 1 uM 4HT in all cultures, (Figure 1A). Cell cycle analysis was performed on cultures of F22 cells 72 hours after induction of SMARCB1 protein expression. In SMARCB1 expressing cells 41.4+/−5% of cells were arrested in G_0_ compared with 7.0+/−1.4% in un-induced cells (Supplementary Figure S1A), consistent with observations made previously that SMARCB1 can induce cell cycle arrest [14]–[16]. In contrast, STMpc cells induced to express SMARCB1 showed little change in the cell cycle (Supplementary Figure S1A). One explanation for this is that the parental line, STM91-01, is derived from a RT lung metastasis and may have acquired additional oncogenic mutations and hence greater resistance to growth inhibitory signals. Reincke et al [21] also found this cell line difficult to transduce with recombinant adenovirus containing SMARCB1 and reportedly found inconsistent cell cycle changes following infection. CDKN1C mRNA was elevated in all lines (F22, F13, STMpc) following induction of SMARCB1 expression with 1 uM 4HT whereas other CDK family members including CDKN1A (p21/WAF1) and CDKN1B (p27) did not show uniform responses following 4HT treatment (Figure 1B). Quantitative analysis of CDKN1C expression by real-time PCR in F22 cells showed four-fold increases in CDKN1C expression 24 hours following SMARCB1 induction, and STMpc showed six-fold increases in CDKN1C expression (Figure 1C). 1 uM 4HT alone had a negligible effect on CDKN1C expression in parental G401 and STM91-01 cultures demonstrating that the observed effect on CDKN1C expression was directly attributable to the induction of SMARCB1 protein (results not shown). Endogenous levels of CDKN1C protein were low in un-induced F22 cells however following SMARCB1 induction, were increased to levels consistent with the proportional increase in CDKN1C transcript levels (Figure 1D).

### Enforced expression of CDKN1C arrests proliferation in G401 cells and CDKN1C knock-down increases proliferation and attenuates SMARCB1-induced proliferation arrest

**Figure 2:**
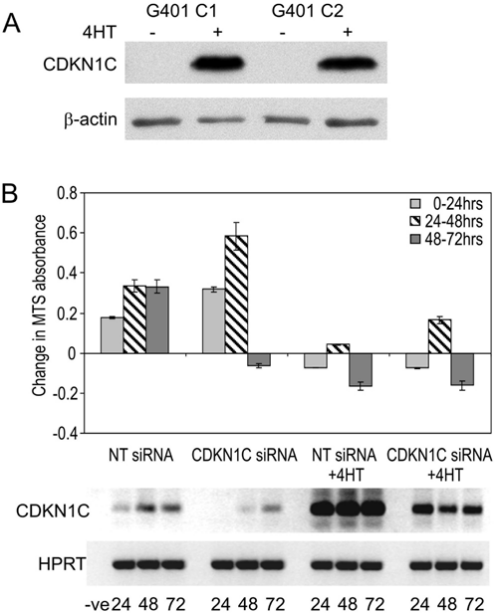
CDKN1C levels modulate growth in rhabdoid tumor cells. (A) Western blot showing induction of CDKN1C protein in G401 clones C1 and C2. Western blotting was performed with the p57 (C20) antibody (Santa Cruz Biotech) with an exposure time of 30 seconds. (B) MTS proliferation assay and CDKN1C RT-PCR following siRNA treatment in F22 cells. The bar graph represents the absorbance difference at each time interval. The data shown is the mean of five independent experiments and the error bars represent the standard error of the mean. CDKN1C siRNA leads to increased proliferation in F22 cells and attenuated proliferation arrest in the 24 to 48 hour time interval in F22 cells induced to express SMARCB1. These effects were positively correlated with the levels of CDKN1C expression following CDKN1C siRNA transfection determined after 35 PCR cycles (lower panel).

To examine the direct effect of induced CDKN1C expression in rhabdoid tumor cells, G401 clones over-expressing full-length human CDKN1C under the control of the tamoxifen inducible promoter (pcDNA3 UAS CDKN1C and pEFpuro/GEV), were selected in geneticin and puromycin. Induction of CDKN1C protein in G401 cells induced cell cycle arrest (Figure 2A and Supplementary Figure S1B). Endogenous CDKN1C protein was not detectable by Western blotting at the short exposure time-setting used to detect over-expression of CDKN1C. In clone G401 C1, expressing the highest level of CDKN1C protein, 24.7+/−1.67% of cells were arrested in G_0_, compared with 2.3+/−0.14% in un-induced cultures (Supplementary Figure S1B). To further ascertain the functional significance of CDKN1C induction in RT cells, a CDKN1C siRNA pool was used to knock-down the low level of endogenous CDKN1C in F22 cells, and to knock-down CDKN1C mRNA induced following SMARCB1 re-expression. CDKN1C siRNA transfection in F22 cells eliminated CDKN1C expression and led to increased proliferation, as measured by a greater positive change in MTS absorbance over the first 48 hours following transfection, as compared with F22 cells treated with a non-targeting control siRNA (Figure 2B). CDKN1C siRNA also attenuated the effect of SMARCB1 induction on restricting cell proliferation. Cells in which SMARCB1 expression was induced with 1 uM 4HT by 24 hours and transfected with CDKN1C siRNA, exhibited a significant and reproducible positive increase in MTS absorbance between 24 and 48 hours, reflecting an increase in the number of proliferating cells in the culture when compared with cells transfected with non-targeting siRNA +4HT. This correlated with reduced levels of CDKN1C mRNA after 48 hours in the +4HT CDKN1C siRNA-treated cultures. Between 48 and 72 hours, cells in the 4HT and non-targeting siRNA, CDKN1C siRNA, and CDKN1C siRNA and 4HT cultures, showed a reduction in the number of viable cells present. In the CDKN1C siRNA culture this likely reflects a reduction in the rate of cell proliferation as the cells approach senescence. In the cultures treated with non-targeting siRNA and 4HT, and CDKN1CsiRNA and 4HT, the decrease in cell viability probably reflects the long-term effect of sustained SMARCB1 expression on cell viability. This may suggest that SMARCB1 modifies the expression of other genes in addition to CDKN1C that affect cell proliferation and viability. Nevertheless these results clearly show that CDKN1C is an important modulator of G401 growth as small changes in CDKN1C expression affect proliferation, and furthermore provide evidence for parallels between the actions of SMARCB1 and CDKN1C in regulating RT proliferation.

### SMARCB1 expression causes increased histone acetylation at the *CDKN1C* promoter

To investigate the mechanism associated with the SMARCB1-induced upregulation of CDKN1C in RT cells, a region within the 5′CpG island spanning the *CDKN1C* transcription start site, from −41 to +114 was analysed for changes in methylation, pre and post SMARCB1 induction. Methylation of this region has previously been shown to correlate with silencing CDKN1C expression in cancer cell lines [22]. Bisulphite sequencing on 10 clones pre and post induction with 4HT revealed that the *CDKN1C* promoter was largely unmethylated in un-induced F22 and STMpc cells and remained unmethylated following SMARCB1 induction, (Supplementary Figure S2). Furthermore, treatment of parental G401 and STM91-01 cells with 2 uM 5aza2dC had no significant effect on CDKN1C expression consistent with a lack of promoter methylation.

**Figure 3:**
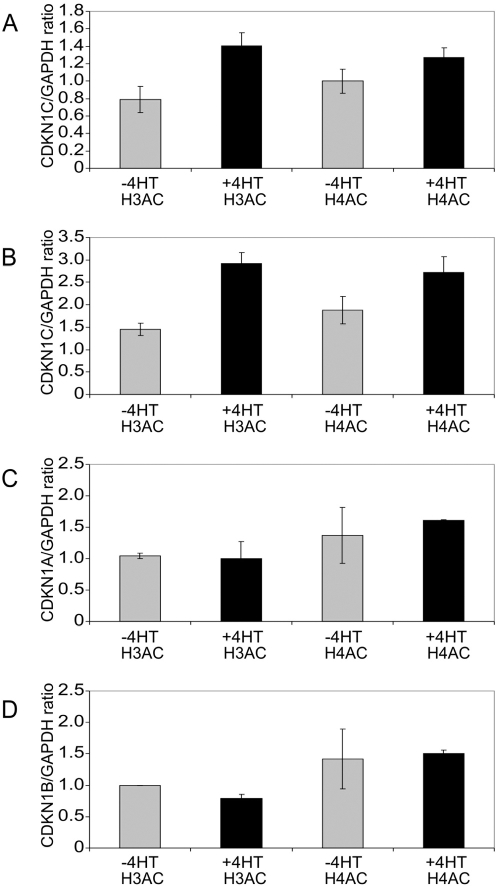
SMARCB1 increases histone acetylation at the CDKN1C promoter. (A) Acetylation of histone H3 and histone H4 at the *CDKN1C* promoter in un-induced (−4HT) and in induced (+4HT) F22 cells. (B) Acetylation of histone H3 and histone H4 at the *CDKN1C* promoter in un-induced (−4HT) and in induced (+4HT) STMpc cells. CDKN1C signal was normalized to GAPDH. Acetylation at histone H3 and H4 was increased at the *CDKN1C* promoter in both F22 and STMpc cells. (C) Acetylation of histone H3 and histone H4 at the *CDKN1A* promoter. (D) Acetylation of histone H3 and histone H4 at the *CDKN1B* promoter. CDKN1A and CDKN1B signal was normalized to GAPDH. Error bars represent the standard error of the mean and were derived from three independent experiments.

We then performed *CDKN1C* promoter chromatin immunoprecipitation (ChIP) with antibodies to acetylated histones H3 and H4, 48 hours post SMARCB1 induction. Quantitative PCR was performed on recovered DNA spanning the *CDKN1C* transcription start site from −72 to +89 and normalized to GAPDH [22]. Three independent ChIP analyses each, on both F22 and STMpc rhabdoid tumor cell lines, showed that the *CDKN1C* promoter is acetylated in un-induced F22 and STMpc cells, however histone H3 acetylation increased 2 fold in both cell lines following SMARCB1 induction with 4HT. A less marked increase in histone H4 acetylation was observed in both cell lines cells (Figure 3A and B). Histone acetylation was also examined at the *CDKN1A* and *CDKN1B* promoters in F22 cells. In contrast to acetylation of the *CDKN1C* promoter, histone H3 acetylation was either unchanged (*CDKN1A* promoter) or slightly decreased (*CDKN1B* promoter) following SMARCB1 induction, and histone H4 acetylation was not significantly changed at either promoter (Figure 3C and D). These results show that SMARCB1 specifically acetylates histones H3 and H4 at the *CDKN1C* promoter either directly, or indirectly through the recruitment of histone acetyltransferases (HATs) or through inhibition of histone deacetylases (HDACs).

### *CDKN1C* is upregulated in RT cells by HDACi and promoter histone acetylation is increased

**Figure 4:**
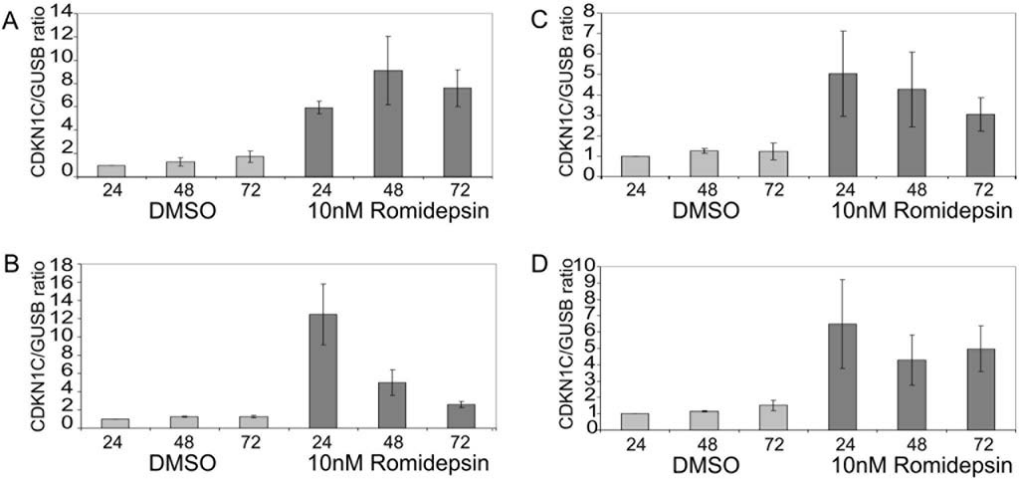
Romidepsin increases CDKN1C expression in rhabdoid tumor. CDKN1C expression normalized to the GUSB control gene in (A) G401 (B) STM91-01 (C) SJSC and in (D) BT16 cells treated with 10 nM Romidepsin. All data represent the mean of three independent experiments and the error bars represent the standard error of the mean.

**Figure 5:**
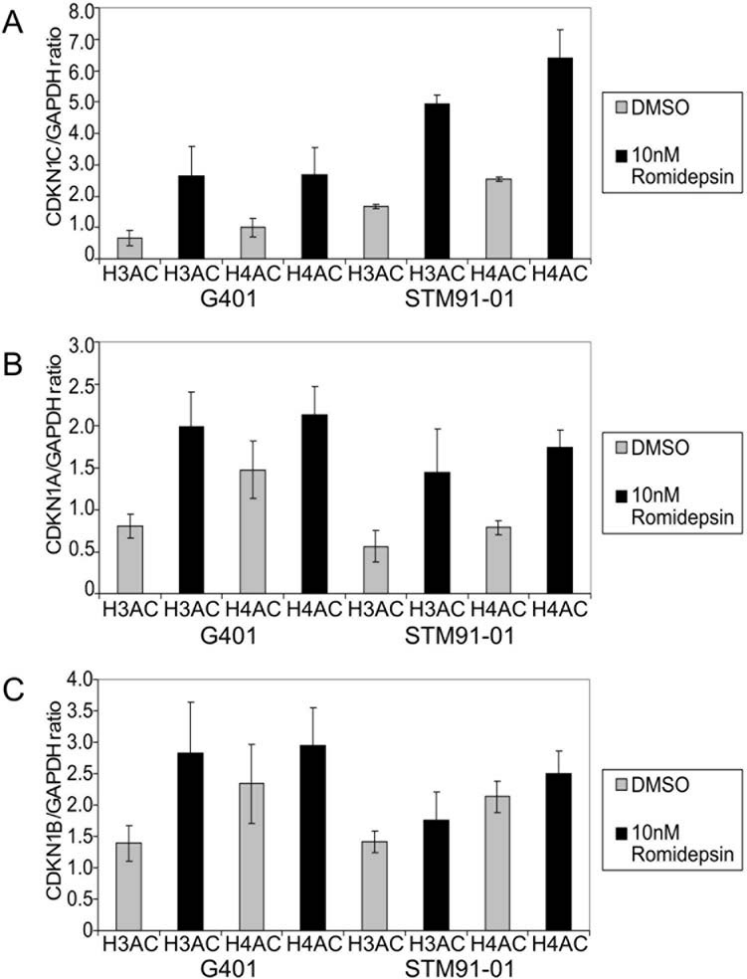
Romidepsin increases histone acetylation at the CDKN1C promoter. (A) Acetylation of histone H3 and histone H4 at the *CDKN1C* promoter in G401 and in STM91-01 cells treated with 10 nM Romidepsin and in control cells treated with 0.01%DMSO. Acetylation at both histone H3 and histone H4 was significantly increased in both treated cultures. (B) Acetylation of histone H3 and histone H4 at the *CDKN1A* promoter in G401 and in STM91-01 cells treated with 10 nM Romidepsin and in control cells treated with 0.01%DMSO. Histone acetylation is increased in both cell lines following Romidepsin treatment although the increases are not as marked as those seen at the CDKN1C promoter following Romidepsin treatment. (C). Acetylation of histone H3 and histone H4 at the *CDKN1B* promoter in G401 and in STM91-01 cells treated with 10 nM Romidepsin and in control cells treated with 0.01%DMSO.

Romidepsin is a potent broad-spectrum inhibitor of class 1 and class II histone decetylases (reviewed in [23]). We reasoned that HDACi might mimic the effect of SMARCB1 on *CDKN1C*. CDKN1C expression was significantly increased in all RT cell lines including G401, STM91-01, SJSC and BT16 lines following treatment with 10 nM Romidepsin for 24 hours (Figure 4A to D). Levels of CDKN1C mRNA induction quantified by real-time PCR varied from six-fold in BT16 cells to twelve-fold in STM91-01 cells. After 48 hours culture with 10 nM Romidepsin, cells showed signs of apoptosis. This coincided with a decrease in the levels of the GUSB house-keeping gene therefore the levels of CDKN1C induction at 24 hours most reliably reflect the true effect of Romidepsin on CDKN1C expression. The levels of CDKN1C induction at 24 hours in G401 and STM91-01 cells treated with Romidepsin slightly exceeded those induced with SMARCB1. Cell cycle analysis was performed on G401 and STM91-01 cells treated with 1 nM Romidepsin after 72 hours. In both cell lines, this treatment led to a significant increase in the percentage of cells in G_0_ (Supplementary Table S1). This was more pronounced in G401 cells compared with STM91-01 cells. In control G401 cells treated with 0.01% DMSO, 4.1+/−0.35% of cells were in G_0_ compared with 19.3+/−1.6% in cultures treated with 1 nM Romidepsin. In STM91-01, 9.3+/−1.9% of cells treated with 0.01% DMSO were in G_0_ compared with 16.7+/−2.0% treated with 1 nM Romidepsin. *CDKN1C* promoter ChIP analysis was performed in G401 and STM91-01 post treatment with 10 nM Romidepsin for 48 hours. Both histone H3 and H4 acetylation was significantly increased at the *CDKN1C* promoter in treated cells, consistent with the increase in CDKN1C expression, (Figure 5A). Increases in promoter histone acetylation were also observed for *CDKN1A* however, these were not as pronounced as the histone acetylation observed for *CDKN1C* (Figure 5B). In contrast, CDKN1B did not show significant increases in promoter histone acetylation following Romidepsin treatment (Figure 5C).

### *CDKN1C* allelic expression is modulated in RT cells with reconstituted expression of SMARCB1 and with HDACi

**Figure 6:**
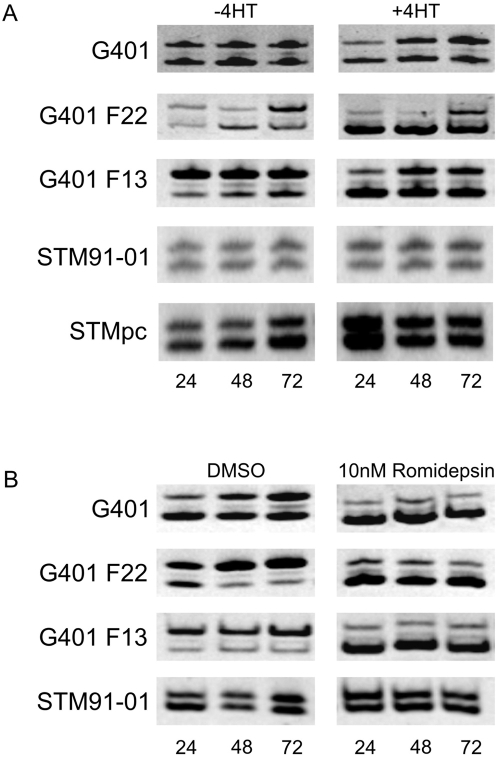
SMARCB1 and HDACi have identical effects on allelic expression of CDKN1C. (A) *CDKN1C* allelic expression in un-induced and in induced cultures of G401, F22, F13, STM91-01 and in STMpc cells. Changes in the pattern of allelic expression can be seen in F22 and in F13 cells following induction of SMARCB1 expression with 1 uM 4HT. Wild-type G401 cells did not show any change in allelic expression following 4HT treatment. Biallelic *CDKN1C* expression persisted in STM91-01 cells and in STMpc cells. (B) *CDKN1C* allelic expression in un-induced cultures of F22 and F13 with 0.01% DMSO and 10 nM Romidepsin and in G401 cells. Changes in allelic expression can be seen with Romidepsin treatment in F13, F22 cells and in wild-type G401 cells. As with SMARCB1 induction in STMpc cells, allelic expression in STM91-01 cells was unchanged following Romidpesin treatment.

To examine *CDKN1C* imprinting, we took advantage of deletion polymorphisms within *CDKN1C* exon 1 in G401 and in STM91-01 cells, [24], [25]. *CDKN1C* alleles differing in size by 24 bp and 12 bp were identified in G401 and STM91-01 cells respectively (Supplementary Figure S3). Both cell lines exhibited biallelic expression, suggesting loss of imprinting. This is in contrast to *CDKN1C* imprinting in the normal kidney where the gene shows monoallelic expression [26]. The effect of SMARCB1 expression on *CDKN1C* imprinting in G401 clones F13 and F22 and in the STM91-01 pooled cells, STMpc, was examined. Induction of SMARCB1 protein in F22 cells led to the restoration of monoallelic CDKN1C expression with expression predominantly from the short allele 24 hours post induction, and a trend towards expression from the short *CDKN1C* allele in the weaker SMARCB1 expressor, F13 (Figure 6A). 4HT alone had no sustained effect on the pattern of allelic expression in parental G401 cells, demonstrating specificity for the SMARCB1 effect. A variable dominant expression from the long allele was sometimes observed in different wild-type and recombinant un-induced G401 cells reflecting the unstable nature of *CDKN1C* imprinting in these cultures. The short allele was however always reproducibly expressed in replicate cultures following SMARCB1 induction in the G401 background suggesting that SMARCB1 expression leads to stabilization of the *CDKN1C* imprint and promotes monoallelic expression. In contrast, induction of SMARCB1 expression in STMpc cells did not alter biallelic expression of CDKN1C (Figure 6A). We then examined allelic expression in response to treatment with 0.3 uM TSA and 10 nM Romidepsin and observed a shift to the short allele mimicking the effect of SMARCB1 induction. This was most marked with 10 nM Romidepsin (Figure 6B). The parental G401 line was also sensitive to TSA and Romidepsin, and the direction of monoallelic expression following treatment was identical to that observed in F22 and F13 cells (Figure 6B). Consistent with the response to SMARCB1 induction, TSA and Romidepsin had no effect on the pattern of allelic expression in STM91-01 cells (Figure 6B). Taken together these results show that SMARCB1 over-expression, and HDACi drugs, have identical affects on the allelic expression of *CDKN1C*.

Imprinting at *CDKN1C* is typically, although not exclusively, regulated via maintenance of allele specific patterns of methylation at the adjacent 11p15.5 Imprinting Centre 2 (*IC2*) involving antisense transcription of the non-coding RNA, LIT1 [27], [28]. Mosaic loss of methylation at IC2 may lead to low levels of biallelic CDKN1C expression. However, in both G401 and STM91-01 cells, normal methylation at *IC2* was observed suggesting that an alternative mechanism is associated with loss of *CDKN1C* imprinting in RT cell lines ( Supplementary Figure S4). Other RT cell lines including SJSC and BT16 (not informative for *CDKN1C* imprint analysis) also maintained normal methylation at *IC2* (results not shown).

### CDKN1C expression is absent in rhabdoid tumor specimens

The relevance of impaired CDKN1C expression in RT cell lines to the aetiology of RT was further ascertained by examination of clinical rhabdoid tumor specimens. Tumors classified as rhabdoid or ATRT on the basis of identified *SMARCB1* mutations, absence of SMARCB1 protein expression or histological criteria, were examined for CDKN1C mRNA expression by RT-PCR and for CDKN1C protein by immunohistochemistry. 6/6 tumors with viable mRNA showed negligible or absent CDKN1C expression after 45 cycles of RT-PCR, although CDKN1A and CDKN1B expression persisted in these tumors, (Supplementary Figure S5A). All tumors examined by immunohistochemistry (n = 12) lacked nuclear staining for CDKN1C and showed negligible cytoplasmic staining (Table 1 and Supplementary Figure 5B). A subset of RT show loss of CDKN1C protein even in the presence of detectable wild-type *SMARCB1* suggesting that additional genetic factors may contribute to *CDKN1C* silencing in rhabdoid and related tumors. Four tumor specimens, with confirmed SMARCB1 mutations, and lacking CDKN1C expression, exhibited methylation indices at *IC2* within the normal range (results not shown) suggesting that epigenetic change at *IC2* is not responsible for *CDKN1C* silencing in primary rhabdoid tumors, consistent with observations in RT cell lines. There was insufficient DNA available from SMARCB1 expressing tumors for IC2 methylation analysis by southern blotting, and it is unclear whether imprinting defects at IC2 contribute to the absence of CDKN1C expression within this sub-set.

**Table 1:** Molecular genetic features of ATRT.

Tumor	Mutation	Prediction	SMARCB1 protein	Tumor site	22qLOH	CDKN1C mRNA	CDKN1C protein
1502	None		Yes	CNS	No	NA	No
1552	None		Yes	CNS	No	NA	No
1595	None		Yes	Renal	No	NA	No
1758	None		Yes	Posterior fossa	Yes	NA	No
1877	c118C>T (exon 2)	ARG40STOP	No	abdominal	Yes	No	No
1918	None		No	Chloroid plexus	Yes	NA	No
1938	c1144delG (exon 9)	Frame shift	No	CNS	Yes	faint	No
1993	c325insG (exon 3)	Frame shift	No	CNS	Yes	faint	No
3022	None		Yes	Renal	Yes	No	No
3074	Homozygous deletion of gene	No protein made	No	CNS	Yes	No	No
3161	c601C>T (exon 5)	ARG201STOP	No	CNS	No	faint	No
	c795+2 indel ATGA	Splice donor mut^n^ removes exon 6					
3180	c157C>T (exon 2)	ARG53STOP	No	CNS	No	NA	No
	c569–570 ins18 (exon 5)	Frame shift					

Table 1 summarizes the molecular genetics of clinical specimens of ATRT. In tumor 3074 *SMARCB1* was homozygously deleted. In tumors 3161 and 3180 two independent *SMARCB1* mutations were identified. NA; tumor RNA was not available.

## Discussion

In this study we identify the imprinted cell cycle regulator, *CDKN1C*, as a new and important SMARCB1 target in RT and furthermore demonstrate that SMARCB1 has the capacity to restore the expression of genes that are epigenetically silenced, consistent with its proposed role in chromatin remodelling. We show evidence that the mechanism by which CDKN1C expression is induced by SMARCB1 is via increased promoter histone H3 and H4 acetylation and that the HDACi, Romidepsin, activates CDKN1C expression in a related manner, leading to cell cycle arrest in RT cell lines. The parallel effects of SMARCB1 and HDACi, in association with an absence of CDKN1C expression in clinical specimens of rhabdoid tumor, and the demonstrated sensitivity of RT cell lines to small changes in CDKN1C expression mediated via siRNA, suggest that loss of CDKN1C is integral to the aetiology of RT, and furthermore that RT may be responsive to specific HDACi that increase endogenous levels of CDKN1C. This study reveals new insight into the molecular aetiology of malignant RT.

Cell cycle regulators have been previously studied in RT cell lines. SMARCB1 is a transcriptional co-activator of *p16^INK4a^* and is required to bring the BRG1 chromatin remodelling complex to the *p16^INK4a^* promoter. Activation of *p16^INK4a^* inhibits the Cyclin D-CDK4 complex with the result that pRB is retained in its anti-proliferative hypo-phosphorylated state and cells arrest in G1 [15], [16], [29]. pRB is the critical endpoint for G1 arrest in RT. The CIP KIP family member *CDKN1A* is a critical effector of signal transduction pathways and has also previously been implicated as a downstream target for SMARCB1 in RT cell lines [14]. CIP KIP inhibitors bind and inhibit the kinase activity of CDK-cyclin complexes including Cyclin A-CDK2 and Cyclin E-CDK2 through a common shared N-terminal domain. *CDKN1A* is up-regulated following re-expression of SMARCB1, and expression is coincident with G1 arrest in these cells [14]. Cyclin E can overcome SMARCB1 induced proliferation arrest in RT cell lines also implying involvement of CIP KIP inhibitors in rhabdoid tumor, however in other studies CDKN1A and CDKN1B expression were unchanged by SMARCB1 expression [16]. We identified minor transcriptional upregulation of CDKN1A or CDKN1B in two different RT cell backgrounds following transfection with SMARCB1 however, expression of these genes persisted in clinical specimens of ATRT with inactivated *SMARCB1* arguing against a major role in rhabdoid tumor aetiology.

*CDKN1C* is a third member of the CIP KIP family. *CDKN1C* is regulated by p73 during mitotic exit and re-entry into G1 in T98G glioma cells and in myogenic differentiation, *CDKN1C* expression is induced by p73 and p63 to maintain pRB in an active hypophophorylated state [30]. *CDKN1C* is not transactivated by p53 [31]. Our results therefore suggest that SMARCB1 could be part of a pathway involving p73 or alternatively that SMARCB1 independently regulates *CDKN1C*. Due to a lack of suitable SMARCB1 antibodies for immunoprecipitation, we were unable to perform ChIP experiments to show whether SMARCB1 transactivated CDKN1C by direct promoter binding.

CDKN1C is expressed in human fetal brain and has been implicated in regulating the migration of neurons entering the neural plate and in the later stages of neurogenesis in driving the differentiation of oligodendrocyte precursor cells [32], [33]. CDKN1C is also not expressed in human astrocytomas however re-expression leads to a G1 block associated with hypophosphorylation of pRB, consistent with a tumor suppressor role [34]. Significantly and perhaps not surprisingly we found that CDKN1C expression was absent from the clinical tumor specimens we examined, of which the majority were CNS AT/RT, and our evidence from RT cell lines also supports an important role for CDKN1C in RT growth suppression.

Silencing of *CDKN1C* in cancer is usually epigenetic and associated with promoter methylation or histone deacetylation [22]. In gastric tumors both mechanisms silence *CDKN1C* and expression can be restored by demethylating reagents in cases where the promoter is hypermethylated or by HDACi in cases where promoter histones are deacetylated [35]. Growth suppression in *CDKN1C*-transduced leukaemia cell lines is dependent on the extent of endogenous *CDKN1C* promoter methylation within the recipient cell background [36]. Furthermore aberrant methylation of the cell cycle control pathway including *p73*, *p15* and *CDKN1C* defines a subset of ALL patients with poor prognosis [37]. Evidence also exists for *CDKN1C* silencing in lung and breast cancer by promoter methylation [38]. Our results, showing increases in histone acetylation at the *CDKN1C* promoter in RT cell lines, following treatment of the cells with Romidepsin and following SMARCB1 induction, suggest that *CDKN1C* is epigenetically silenced in RT by histone deacetylation at the promoter, and that SMARCB1 is complexed with histone acetyl transferases or that it may have instrinsic acetylation properties. The promoters of *CDKN1A* and *CDKN1B* were not significantly affected by SMARCB1 induction and these genes exhibited minor changes in expression. In contrast, CDKN1C expression was markedly increased and this coincided with a specific increase in histone H3 acetylation and less marked increase in histone H4 acetylation. The predominant acetylation at histone H3 is consistent with studies showing that the transcriptionally active murine *cdkn1c* promoter is associated with acetylated histone H3 [39].

*CDKN1C* is a paternally imprinted gene and in most normal tissues is expressed from the maternal allele although frequently leaky expression also occurs from the paternal allele. SMARCB1 over-expression and HDACi did not universally induce monoallelic transcription of *CDKN1C* in all cell lines suggesting that SMARCB1 cannot be involved in establishing imprinting at *CDKN1C*, but that it is able to recognize and induce transcription from appropriately imprinted alleles. Significantly LIT1 methylation was normal in RT cell lines and in RT with loss of SMARCB1 function, demonstrating that loss of CDKN1C imprinting must involve mechanisms independent of IC2 on 11p15.5.

*Cdkn1c* has been recently shown to be a critical regulator of embryonic growth and is the only CIP KIP protein that is imprinted and essential for normal development [40]. 50% of patients with the overgrowth disorder Beckwith Wiedemann syndrome commonly have epigenetic silencing of *CDKN1C* caused by a methylation defect within *IC2* on 11p15.5. These patients are at risk for cancer in early childhood. The spectrum of tumors in *IC2* imprinting defect cases includes hepatoblastoma, rhabdomyosarcoma and gonadoblastoma [41] although these affect fewer than 5% of these patients. Jackson et al [42] recently reported a case with BWS and an *IC2* defect with ATRT. However the ATRT also bore somatic loss of 22q and an inactivating *SMARCB1* mutation, demonstrating that germ-line inactivation of *CDKN1C* was not sufficient to cause ATRT. This is consistent with the widespread inactivation of *CDKN1C* in many different cancers with additional oncogenic mutations and suggests that *CDKN1C* silencing contributes, but is alone insufficient, to cause tumors. Germline *SMARCB1* mutations have been recently described in rare families with predisposition to RT and to schwannomatosis [5], [12], [13]. It might be expected that individuals carrying inactivating mutations in *SMARCB1* in the germline might display phenotypic features in line with impaired CDKN1C expression such as high birth weight. However this has not been reported in the literature and too few cases have been identified to date to directly examine these associations.

The fact that epigenetic silencing of *CDKN1C* is so widespread in human cancer makes it an attractive target for epigenetic therapies. To our knowledge it has not been previously identified as a target for Romidepsin. The promoter of *CDKN1A* is however frequently acetylated by HDACi and expression is induced (reviewed in [43]). The fact that *CDKN1C* is an imprinted gene makes it a more attractive target as its imprinted status provides an additional layer of developmental regulation and implies that comparatively small changes in expression in embryonic-like cells are likely to have potent effects on cell proliferation and differentiation [40]. In addition it has recently been shown to be a critical regulator of embryonic growth regulating through IGF1, a growth factor frequently abnormally expressed in human cancer.

In summary we have demonstrated that *CDKN1C* is an important target for SMARCB1 and that RT cells are sensitive to small changes in CDKN1C expression. We show for the first time that SMARCB1 has a role in *CDKN1C* promoter histone acetylation suggesting opportunities for further investigation of signalling pathways that may be epigenetically regulated by *SMARCB1*. Furthermore as Romidepsin restores *CDKN1C* promoter histone acetylation, leading to increased CDKN1C expression, we propose that Romidepsin and related compounds could be further explored as therapeutic reagents in the treatment of RT through restoration of gene expression patterns lost as a result of SMARCB1 inactivation.

## Materials and Methods

### Cell lines and cell culture

The G401 RT cell line was obtained from the American Type Culture Collection (ATCC). The establishment of STM91-01, BT16 and SJSC were previously reported [44], [45]. These RT cell lines were maintained in RPMI 1640 supplemented with 10% fetal calf serum (FCS) and grown at 37°C in 5%CO_2_. *SMARCB1* recombinant STM91-01 cells were grown in the same media supplemented with 100 µg/mL hygromycin (Roche, Mannheim, Germany) and 1 µg/mL puromycin (Sigma Chemical Company, St. Louis, MO). G401 cells were maintained in McCoy's 5A modified media supplemented with 10% FCS, and both *SMARCB1* and *CDKN1C* recombinant G401 clones were grown in the same media supplemented with 600 µg/mL geneticin (Invitrogen, Groningen, Netherlands) and 5 µg/mL puromycin (Sigma).

Induction of SMARCB1 and CDKN1C in transformed cell lines was achieved by the addition of 1 µM 4-hydroxytamoxifen (4HT: Sigma Aldrich Castle Hill Australia). 4HT (1 mM) and Trichostatin A (TSA; Sigma Aldrich) (3.0 mM) were prepared in 100% ethanol. Romidepsin (10 mM)(Gloucester Pharmaceuticals) and 5-Aza-2′-deoxycytidine (5aza2dC; Sigma Aldrich) (2 mM) were prepared in 100% DMSO and stored in aliquots at −20°C. Appropriate dilutions were made into culture medium for experimental analyses. Relevant solvent controls were included in all experiments. Culture media and reagents were replaced every 24 hrs.

For imprinting and gene expression assays, cells were plated as replicate cultures at 2×10^5^ cells/well in 2 ml culture volumes. Cells were trypsinized at 24 hourly time points, over a total time-course of 72 hours, washed in PBS, and pellets were frozen at −80°C for future RNA and DNA extraction.

### Southern blotting

To confirm *SMARCB1* deletion or rearrangement in RT cell lines and in clinical specimens of rhabdoid tumor, 10 ug of RT cell line genomic DNA was digested with a panel of restriction enzymes and run overnight on 0.8% agarose gels alongside normal human genomic DNA as a control. DNA was transferred overnight in 20× SSC buffer onto Zeta Probe ^R^ GT Genomic tested blotting membrane (Biorad, Hercules CA 94547). Membranes were fixed under UV light, prehybridized at 60°C in ExpressHyb buffer (Clontech, Mountain View CA 94043) and hybridized with a ^32^P-labelled full length *SMARCB1* PCR product, prepared by the random priming method, for 18 hours. Membranes were washed at room temperature with 2× SSC / 0.05% SDS for 40 mins and then with 0.1× SSC / 0.1% SDS for a further 40 mins at 55°C and exposed to phosphoimager screens for 24 to 48 hours. Bands were visualized with ImageQuant TL v2003.02 software following digital capture on a scanning phosphoimager (Molecular Dynamics).

### Cell cycle analysis

5×10^5^ cells were seeded and treated for 72 hrs. Media was replaced each 24 hrs. 5×10^5^ cells were stained for cell cycle analysis using a modified flow cytometry assay [46]. Briefly, 5×10^5^ cells were collected, washed in PBS/0.1% FCS, fixed with 1% formaldehyde (Polysciences, Warrington, PA) on ice for 30 mins, then permeabilized on ice with 0.5% Triton X-100 in PBS (Roche) for a further 15 mins. Cells were then incubated on ice in the dark for 45 mins with either PE-conjugated mouse anti-human Ki67 monoclonal antibody or PE-conjugated mouse IgG1 κ monoclonal isotype control (BD Biosciences). After a final wash with PBS/0.1% FCS, cells were resuspended in 0.3 mL of washing buffer containing 1 µg/mL of 7-aminoactinomycin D (7AAD; Molecular Probes, Eugene, USA). Flow cytometric analysis was performed on a LSRII analyser (Becton Dickson) and data analysed using the FACS Diva software program. For each sample, electronic gating was performed on the intact, single cell population. Ki67 expression and 7AAD signal intensity were used to determine the cell cycle status of viable cells.

### Inducible expression system

The human *SMARCB1* coding sequence was PCR amplified from plasmid (PCR4-TOPO-INI1) containing full-length human *SMARCB1*, using forward primer 5′-GGATCCACCATGATGATGATGGCGCTGAGC-3′ and reverse primer 5′-GCCTCGAGTTACCAGGCCGGGGCCGTGTTGG-3′ containing BamH1 and Xho1 restriction sites respectively. The PCR product was digested with BamH1 and Xho1 and inserted into the vector pcDNA3 UAS/neo (kindly provided by Dr.J.Silke, La Trobe University, Victoria, Australia ). The integrity of the construct was verified by direct automated sequencing. The vector was linearized and then co-transfected with a second linearized vector, pEF puro/GEV, into G401 cells. Transfection was achieved using Lipofectamine 2000 (Invitrogen) according to the manufacturer's instructions. Cells containing both pcDNA3 UAS *SMARCB1* and pEF puro/GEV were doubly selected in geneticin and puromycin. Single cells were selected and grown and clones were tested for *SMARCB1* expression by Western blotting following the addition of 1 uM 4HT. The full length human *CDKN1C* coding sequence was PCR amplified from plasmid (pCMV6-XL4 CDKN1C) (Origene Technologies, Rockville, MD), using forward primer 5′ GCGGTACCACCATGTCCGACGCGTCCCTCC-3′ and reverse primer 5′-GCGGATCCTCACCGCAGCCTCTTGCG-3′ containing Kpn1 and BamH1 restriction sites respectively. The PCR product was digested with Kpn1 and BamH1 and inserted into the vector pcDNA3 UAS/neo. Transfection and selection in G401 cells was the same as for *SMARCB1* and clones were tested for CDKN1C expression by western blotting following the addition of 1 uM 4HT.

### Lentiviral production and transfection

The STM91-01 cell line was found to be refractory to transfection using the above vector system and a lentiviral system was used to produce pooled recombinant cell lines [47].

The human *SMARCB1* coding sequence was amplified and cloned into the BamHI and XbaI sites of the vector, pF 5×UAS Sv40 puro, using the same forward primer as above and a modified reverse primer, 5′-GCTCTAGATTACCAGGCCGGGGCCGTGTTGG-3′. HEK 293T cells were transiently transfected with pF 5×UAS Sv40 puro containing SMARCB1or pF GEV16 hygro vector with the packaging vectors pCMVδR8.2 and VSVG, using Effectene (Qiagen, Hilden Germany) as described by the manufacturer. The media was changed after 16 hrs and virus-containing supernatant was collected after 48 hours, filtered through 0.45 µm Millipore filters, and frozen at −80°C.

For transduction in STM91-01 cells, 1×10^6^ of the target cells were washed in PBS and then incubated in virus-containing media containing 5 µg/mL polybrene (Sigma Aldrich) in 6-well plates. Plates were then spun at 2500 rpm for 90 mins at 30°C, incubated for a further 90 mins at 30°C, then returned to 37°C in 5%CO_2_. Media was changed the next day and 2 days after transduction the appropriate selection was added. First, cells were infected with pF GEV16 hygro virus and selected in hygromycin. Hygromycin resistant cells were then infected with pF 5×UAS Sv40 puro/ini1 virus followed by selection in puromycin. A pool of resistant cells was then tested for SMARCB1 expression by western blotting following the addition of 1 uM 4HT.

### siRNA transfection

Optimal transfection conditions for G401 F22 cells were determined using BLOCK-iT fluorescent oligo (Invitrogen). Cells were plated at optimal density for the culture plate used and allowed to adhere overnight. ON-TARGETplus SMARTpool CDKN1C siRNA (Dharmacon, CO, USA) was transfected at a final concentration of 10 nM in the presence of 3 µg/mL DharmaFECT2 transfection reagent (Dharmacon) according to the manufacturer's instructions. Additionally, ON-TARGETplus siCONTROL Non-targeting pool (Dharmacon) was transfected in the same manner as a control (mock) transfection. 6 hrs after transfection, 1 µM 4HT or ethanol control was added to induce SMARCB1 expression. Media was refreshed every 24 hrs, maintaining 1 uM 4HT or ETOH, as required, with 1 nM oligo. Cells were harvested at 24 hr intervals for RNA extraction and for determination of cell proliferation by the MTS assay. CDKN1C expression was examined as described under “Gene expression and imprinting”. Cell proliferation was measured by the MTS-based CellTiter 96 Aqueous non-radioactive cell proliferation assay (Promega, WI, USA). Briefly, triplicate wells of siRNA transfected cells, induced or un-induced for SMARCB1, were incubated with [3-(4,5-dimethylthiazol-2-yl)-5-(3-carboxymethoxyphenyl)-2-(4-sulfophenyl)-2H-tetrazolium, inner salt] (MTS)/phenazine methosulfate (PMS) solution for 2 hrs at 37°C. Well absorbance was measured at 492 nm and a reference wavelength of 620 nm using a Multiskan ES spectrophotometer (Thermo Electron, ON, Canada) at 24 hourly time intervals. The absorbance at each time interval was normalized in each culture to the t_0_ absorbance value, which was set to 1.0. Absorbance differences in each 24 hour interval, reflecting the rate of proliferation in individual cultures, were calculated by subtraction of normalized absorbance values.

### Western blotting

Cell pellets containing at least 2×10^5^ cells were lysed in mammalian lysis buffer (50 mM Tris, pH 7.5; 375 mM NaCl; 1 mM ethylenediamine tetraacetic acid; 1% Triton X-100) and then sonicated to shear DNA. Total protein was determined using the Bicinchoninic (BCA) protein assay (Sigma Aldrich) according to the manufacturer's instructions. Twenty micrograms of protein were separated on 12% sodium dodecyl sulfate-polyacrylamide gels, transferred onto Hybond-P nitrocellulose membrane (Amersham, Buckinghamshire, England), blocked with 5% non-fat dry milk in phosphate-buffered saline (PBS), and probed with the indicated antibodies in 5% non-fat dry milk in PBS with 0.05% Tween-20. HRP-conjugated antibodies were detected using Super-Signal® West Dura Extended Duration Substrate (Pierce, Rockford, IL) and exposing to film (Hyperfilm MP, Amersham). The following antibodies were used for immunoblotting: BAF47 (BD Pharmingen, San Diego, CA) detecting SMARCB1, β-actin (Sigma Aldrich, p57 (C20) (Santa Cruz Biotech, Santa Cruz, CA) or p57 Kip2 (Cell Signaling Technology, Danvers MA USA) detecting CDKN1C, β-actin (Sigma Aldrich), an anti-mouse immunoglobulin-G HRP (Sigma Aldrich) and an anti-goat immunoglobulin-G HRP (Sigma Aldrich) secondary antibody.

### Gene expression and imprinting

Genomic DNA was extracted from RT cell pellets, comprising at least 2×10^5^ cells, using the DNeasy Blood and Tissue kit (Qiagen) according to the manufacturer's instructions. *CDKN1C* primers F4 (5′ GCGTTCTACCGCGAGACG 3′) and R5 (5′ CGAGTGCAGCTGGTCAGCGAG 3′) were used to amplify across the region of deletion polymorphisms in exon 1 using the Enhancer PCR system (Invitrogen Corporation CA 92006) and Hi Fi Taq polymerase (Roche, Mannheim, Germany). PCR products were digested with PvuII and separated on 6% non-denaturing polyacryamide gels containing 10% glycerol to identify polymorphisms required for imprinting analysis as previously described [26]. Bands were visualised by staining with ethidium bromide and digital images were captured following UV exposure on a Biorad Gel Doc 1000 system. PCR products were sequenced using Big Dye terminator technology to determine allele sizes.

RNA was extracted from RT cell pellets comprising at least 2×10^5^ cells, using the RNeasy Mini kit (Qiagen) according to the manufacturer's instructions. 1 ug of RNA was reverse transcribed with random primers (pd(N)6) (Amersham Pharmacia Biotech) and M-MLV Reverse Transcriptase RNaseH minus (Promega Corporation). CDKN1C was amplified with primers F2 (5′GCAGCTGCCTAGTGTCCCGGTC 3′) in exon 1 and R7 (5′ TAAAT TGGCTCACCGCAGCC 3′) spanning exons 2 and 3 within the 3′UTR using the Enhancer PCR system with Hi Fi Taq polymerase. Cycling was 95°C for 3 mins, 45 cycles of 95°C for 45 secs, 56°C for 30 secs, 68°C for 1 min 30 secs and a final extensions step at 68°C for 10 mins. PCR products were digested with PvuII and electrophoresed as described above.

*CDKN1A* (p21) was amplified with primers p21F (5′ GAGGCACCGAGGCACTCAGAG 3′) and p21R (5′ GGCACAAGGGTACAAGACAG 3′) using Go Taq Green master mix (Promega Corporation). Primers were located in exon 1 and 2 respectively to ensure specificity for cDNA. *CDKN1B* (p27) was amplified with primers p27F (5′ CCATGTCAAACGTGCGAGTGT 3′) and p27R (5′ CGTTTGACGTCTTCTGAGG 3′) using Go Taq Green master mix. *CDKN1B* primers were located in separate exons. PCR cycling for *CDKN1A* and *CDKN1B* was at 94°C for 5 minutes, 94°C 30 secs, 55°C for 30 secs, 72°C for 30 secs for at least 28 cycles, followed by a final step at 72°C for 5 minutes.

Real-time Q-PCR for CDKN1C expression was performed using the primer and Taqman probe combination described in Niemitz et al [48]. These were p57 1044F 5′ GCGGCGATCAAGAAGCTG 3′ and p57 1124R 5′ CGACGACTTCTCAGGCGC 3′ with probe p57 1069T FAM 5′ CTCTGATCTCCGATTTCTTCGCCAAGC 3′ TAMRA. Reactions were performed using a Quantitect Probe PCR probe mastermix (Qiagen) and run in triplicate on a Rotorgene 3000 real-time thermal cycler (Corbett Technologies, Sydney Australia). Reaction tubes were set up using the CAS1200 robotic system of Corbett Technologies. Data was analysed using the Rotorgene's comparative quantitation software using selected untreated samples as the calibrator. Expression of the housekeeping gene, glucuronidase beta (GUSB), was examined by Q-PCR using the Quantitect Probe PCR kit (Qiagen) according to the manufacturer's instructions. Reactions were set up and performed in triplicate as for CDKN1C. Data was analysed using comparative quantitation software and untreated samples were used as the calibrator as for CDKN1C. CDKN1C data was normalized to GUSB to derive relative changes in CDKN1C expression. Levels of GUSB remained stable in all reactions, however after 48 hours of treatment with 10 nM Romidepsin, GUSB levels were noted to decline in the RT cell lines.

### Methylation analysis and Chromatin Immunoprecipitation

Methylation at *LIT1* (OMIM 604115) was examined by southern blotting following digestion of 5 ug of DNA with EcoRI and NotI. Southern blots were probed with the probe DMRP recognizing a 4.2 kb methylated and 2.7 kb unmethylated band [27]. Prehybridization, hybridization and washing steps were as described for southern blotting above. Bands were visualized and quantified with ImageQuant TL v2003.02 software following digital capture on a scanning phosphoimager (Molecular Dynamics). The methylation index was calculated as the peak volume of the methylated band divided by the sum of the volumes of the methylated and unmethylated bands after background subtraction.

Methylation within the *CDKN1C* promoter was analysed by sequencing bisulphite-modified DNA.1 ug of DNA extracted from RT cells was bisulphite modified using the MethyEasy™ DNA Bisulphite Modification Kit (Human Genetic Signatures Pty Ltd NSW Australia). Modified DNA easy was amplified using primers p57-CF 5′ GGTTGGGYGTTTTATAGGTTA 3′ and p57-CR 5′ ACCTAACTATCCGATAATAAACTCTTC 3′ generating a 155 bp product derived from the region across the CDKN1C transcription start site from −41 to +114 as described in Kikuchi et al [22]. The amplified product was then cloned into the PCR-Script Amp cloning vector (Stratagene, La Jolla CA). Cloning and transformation were performed as recommended in the PCR-Script™ Amp Cloning kit (Stratagene). Selected blue colonies were grown in liquid media and plasmid DNA was extracted and purified using the QIAprep^R^ Spin Miniprep Kit (Qiagen). Plasmid DNA was sequenced using Big Dye terminator technology (Applied Biosystems) according to the manufacturer's instructions using vector primer T7. At least ten plasmids each were sequenced from un-induced and 4HT-induced RT cultures.

Chromatin immunoprecipitation was performed exactly as described in the protocols provided with the Acetyl-Histone H4 and H3 Immunoprecipitation (ChIP) assay kits (Upstate cell signalling solutions Temecula CA 92590). Sonication was performed by pulsing three times for 10 seconds at 30% power, using a Vibra Cell™ ultrasonic processor (SONICS). Sheared DNA was examined on 1% agarose gels. DNA bound to immunoprecipitated histone was recovered by phenol/chloroform extraction and ethanol precipitation.PCR on ChIP'd DNA was performed across the *CDKN1C* transcription start site from −72 to +89 as described in Kikuchi et al [22]. Primers were p57 ChIP F 5′ GTATAAAGGGGGCGCAGGCGGGCT 3′ and p57 ChIP R 5′ TGGTGGACTCTTCTGCGTCGGGTTC 3′ (product 161 bp). 2 uL of DNA was amplified in a reaction volume of 25 uL containing 1× Sybr Green, 1× Sensimix, 0.25 mM MgCl_2_, and 10 picomoles of each primer as per the recommendations in the 2× Sensimix DNA kit (Quantace, London). Cycling was 95°C for 10 mins, 45 cycles of 95°C for 15 secs, 62°C for 15 secs, 72°C for 20 secs followed by a melt step ramping from 72 to 95°C. GAPDH was amplified using primers GAPDH ChIPF 5′ TCGGTGCGTGCCCAGTTGAACC 3′ and GAPDH ChIPR 5′ ATGCGGCTGACTGTCGAACAGGAG 3′ (product 246 bp) under the same conditions. Reactions were set up in triplicate for each sample on a CAS1200 robotic system and run on a Rotorgene 3000 real-time thermal cycler (Corbett Technologies). Reaction conditions were designed to eliminate primer dimer formation. The comparative quantitation option in the Rotorgene software was used to analyse the data. A normal control DNA sample run in triplicate was used as the calibrator for both CDKN1C and GAPDH. CDKN1C/GAPDH ratios were derived for each run. *CDKN1A* and *CDKN1B* promoters in ChIP fractions were examined in a similar manner using *CDKN1A* promoter primers described in Takai et al [49] and *CDKN1B* promoter primers described in Li et al [50]. These were p21F PROM 5 ′GGTGTCTAGGTGCTCCAGGT 3′ and p21R PROM 5′ GCACTCTCCAGGAGGACACA 3′ (product 255 bp), p27F PROM 5′ GTCCCTTCCAGCTGTCACAT 3′ and p27R PROM 5′ GGAAACCAACCTTCCGTTCT 3′ (product 162 bp). Sensimix reaction mixes were as described for CDKN1C and GAPDH however additional MgCl_2_ was not added. CDKN1A cycling was 95°C for 10 mins, 45 cycles of 95°C for 15 secs, 61°C for 15 secs, 72°C for 20 secs followed by a melt step ramping from 72°C to 95°C. CDKN1B cycling was 95°C for 10 mins, 45 cycles of 95°C for 15 secs, 57°C for 15 secs, 72°C for 20 secs followed by a melt step ramping from 72°C to 95°C. Reaction conditions were designed to eliminate primer dimer formation.

### Tissues and mutation screening in ATRT

Specimens with a clinical diagnosis of RT were submitted to the laboratory for molecular diagnostic testing for *SMARCB1* mutation, or absence of SMARCB1 protein [51], after obtaining informed consent. Analysis of clinical samples was approved by the institutional ethics committee under approval number RCH HREC 24073A. RNA was extracted from frozen sections using the RNeasy mini kit (Qiagen) and DNA was extracted from either frozen sections or formalin-fixed tissue using protocols described in the DNeasy Blood and Tissue kit (Qiagen). cDNA was prepared in a total volume of 40 uL from 1 ug RNA as described above for RT cell lines. Allele loss studies were performed using microsatellite markers mapping to 22q, on both blood and tumor DNA. Markers analysed included *D22S303*, *D22S257*, *D22S301*, *D22S345*, *D22S1685* and *TOPIP2*. Alleles were examined by PCR amplification of 50–100 ng DNA using primers end-labelled with Fam, Hex or Tet. PCR products were electrophoresed on 4.5% 0.2 mm denaturing polyacrylamide gels on an ABI 377 DNA sequencer employing TAMRA 500 (Red) size standard. Sample lanes were tracked with Genescan software Version 3.1.2 and analysed using Genotyper Version 2.1. Mutation screening was performed by direct DNA sequencing of PCR products. Primers were located in introns flanking individual exons and were designed to amplify exonic and flanking splice site sequences. Exon 1 was divided into two separate overlapping fragments for PCR. Primer sequences and reaction conditions are available on request. Sequence analysis was performed by both manual inspection and with Mutation Surveyor (Softgenetics PA 16803). BLAST analysis was performed against genome databases.

### Immunohistochemistry

Immunohistochemical stains were performed on representative formalin-fixed paraffin-embedded tissue sections of a thickness of 2 micron using the monoclonal antibodies INI-1/BAF-47 (clone 25/BAF47, 1∶10, BD Biosciences, North Ryde, Australia) and p57/KIP2 (clone 25B2, 1∶200, Novocastra, Newcastle, UK) [51], [52]. Heat-induced antigen retrieval was performed for 20 minutes at 100°C at pH 6.0 and 9.0, respectively, using target retrieval solution (Dako, Copenhagen, Denmark). The detection system used was standard streptavidin-biotin peroxidase complex (DAKO, Copenhagen, Denmark) with diaminobenzidine as the chromogen. The slides were counterstained with haematoxylin.

## Supporting Information

Figure S1 Cell cycle effects of SMARCB1 and CDKN1C. A. Cell cycle analysis in control cultures and in cultures induced to express SMARCB1. The data represents the mean from three independent experiments and the error bars represent the standard error of the mean. B. Cell cycle analysis in control cultures and in cultures induced to express CDKN1C. The data represents the mean from three independent experiments and the error bars represent the standard error of the mean.
(0.21 MB TIF)

Figure S2 CDKN1C promoter methylation analysis. Allelic bisulphite sequence analysis at the CDKN1C promoter in uninduced (−4HT) and in induced (+4HT) F22 and STMpc cells. Open circles represent unmethylated cytosines and filled circles represent methylated cytosines. No significant change in allelic methylation was identified following the induction of SMARCB1 protein expression.
(0.52 MB TIF)

Figure S3 CDKN1C shows loss of imprinting in rhabdoid tumor. CDKN1C imprinting in G401 and STM91-01 rhabdoid tumor cell lines after 24, 48 and 72 hours in culture. CDKN1C alleles in G401 cells differed by 24 bp and those in STM91-01 differed by 12 bp.
(0.08 MB TIF)

Figure S4 Normal LIT1 methylation is maintained in rhabdoid tumor. Methylation sensitive southern blotting at IC2 (LIT1) in G401 and STM91-01 cells showing normal patterns of methylation, with methylated (4.2 kb) and unmethylated (2.7 kb) bands of similar intensity.
(0.06 MB TIF)

Figure S5 CDKN1C expression in clinical specimens of rhabdoid tumor. A. Gene expression examined by RT-PCR in clinical specimens of rhabdoid tumor. Tumor numbers match those shown in Table 1. Relevant controls are shown: The -ve lane represents the PCR negative control, the RNA -ve control lane represents the negative control for reverse transcription and the RT-ve control represents a control for genomic contamination derived from the induced F22 sample. The positive control in the far right lane shows the level of gene expression in F22 cells expressing SMARCB1. Viability of the cDNA is shown by amplification of the HPRT control gene. The lane for sample 3180 shows that little viable cDNA was obtained from this sample as indicated by the negligible level of HPRT gene expression. B. Immunohistochemical stains for CDKN1C expression. 1) placenta positive control showing strong nuclear staining for CDKN1C, 2) endometrium negative control, 3) placenta without primary antibody 4) tumor section from case 1993 and 5) tumor section from case 1938. 1993 and 1938 show some cytoplasmic staining for CDKN1C but no nuclear staining.
(0.71 MB TIF)

Table S1 Romidepsin reduces proliferation in rhabdoid tumor cells. Table showing the percentage of cells in each cell cycle phase after 72 hours treatment with 1 nM Romidepsin and in controls containing 0.01% DMSO. The G401 data represent the mean values from three independent experiments and the STM91-01 data represent the mean values from six independent experiments. The error represents the standard error of the mean.
(0.03 MB DOC)
