# Supplementary figures and images for: Imprinted CDKN1C Is a Tumor Suppressor in Rhabdoid Tumor and Activated by Restoration of SMARCB1 and Histone Deacetylase Inhibitors
Source: PLoS One. 2009 Feb 16;4(2):e4482. doi: 10.1371/journal.pone.0004482 (PMC2637419; doi:10.1371/journal.pone.0004482)

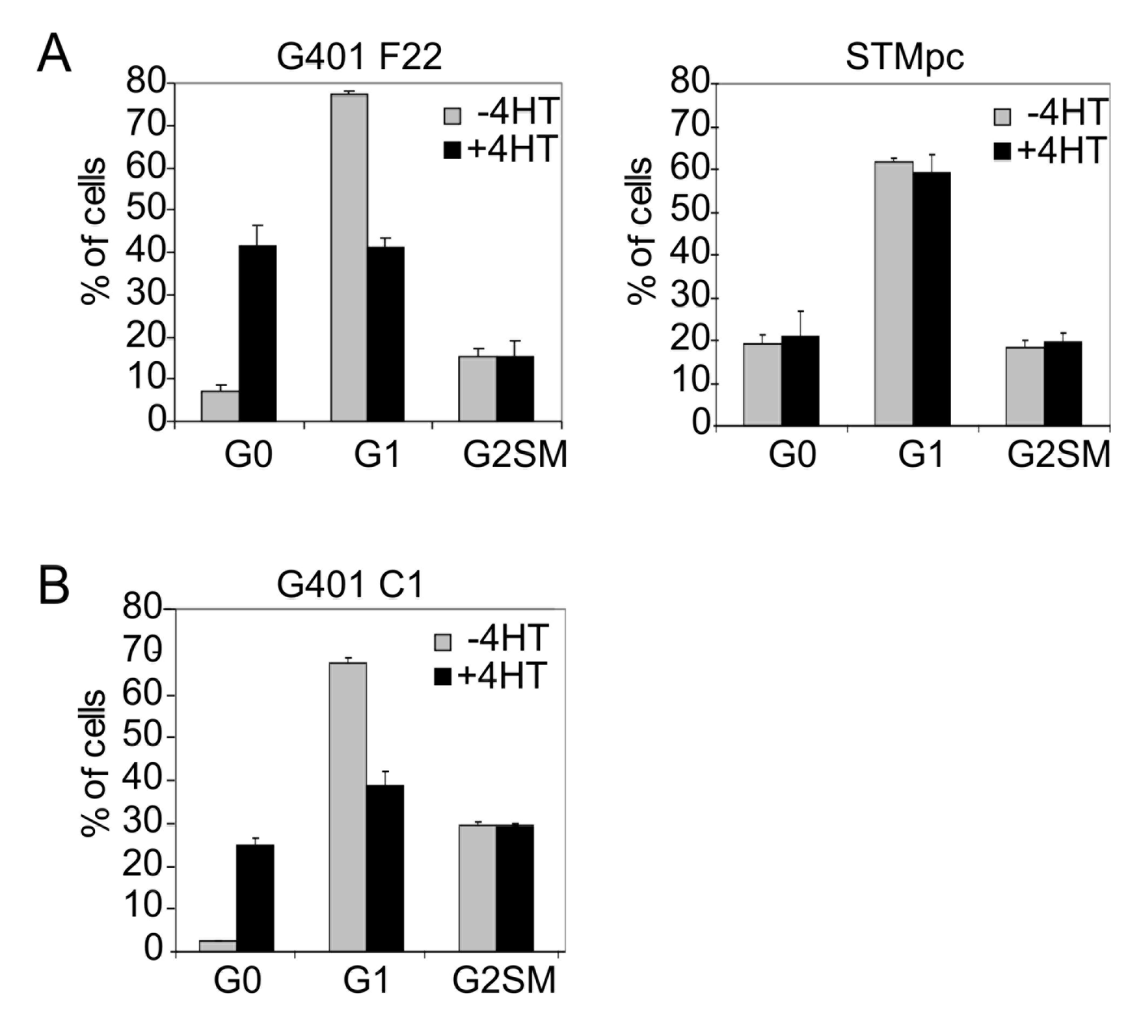

Supplement: Figure S1 — Cell cycle effects of SMARCB1 and CDKN1C. A. Cell cycle analysis in control cultures and in cultures induced to express SMARCB1. The data represents the mean from three independent experiments and the error bars represent the standard error of the mean. B. Cell cycle analysis in control cultures and in cultures induced to express CDKN1C. The data represents the mean from three independent experiments and the error bars represent the standard error of the mean. (0.21 MB TIF) [file pone.0004482.s001.tif]

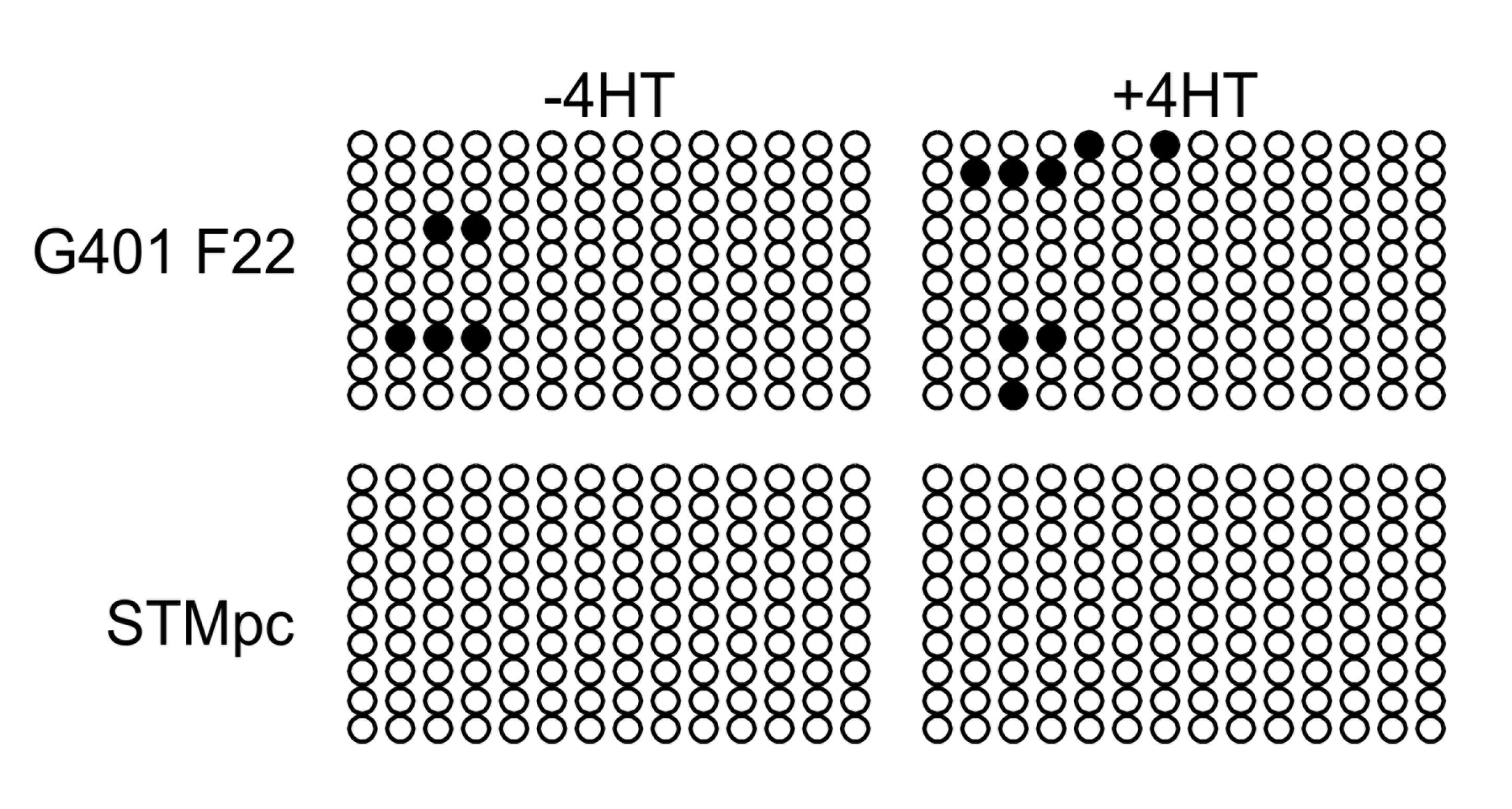

Supplement: Figure S2 — CDKN1C promoter methylation analysis. Allelic bisulphite sequence analysis at the CDKN1C promoter in uninduced (−4HT) and in induced (+4HT) F22 and STMpc cells. Open circles represent unmethylated cytosines and filled circles represent methylated cytosines. No significant change in allelic methylation was identified following the induction of SMARCB1 protein expression. (0.52 MB TIF) [file pone.0004482.s002.tif]

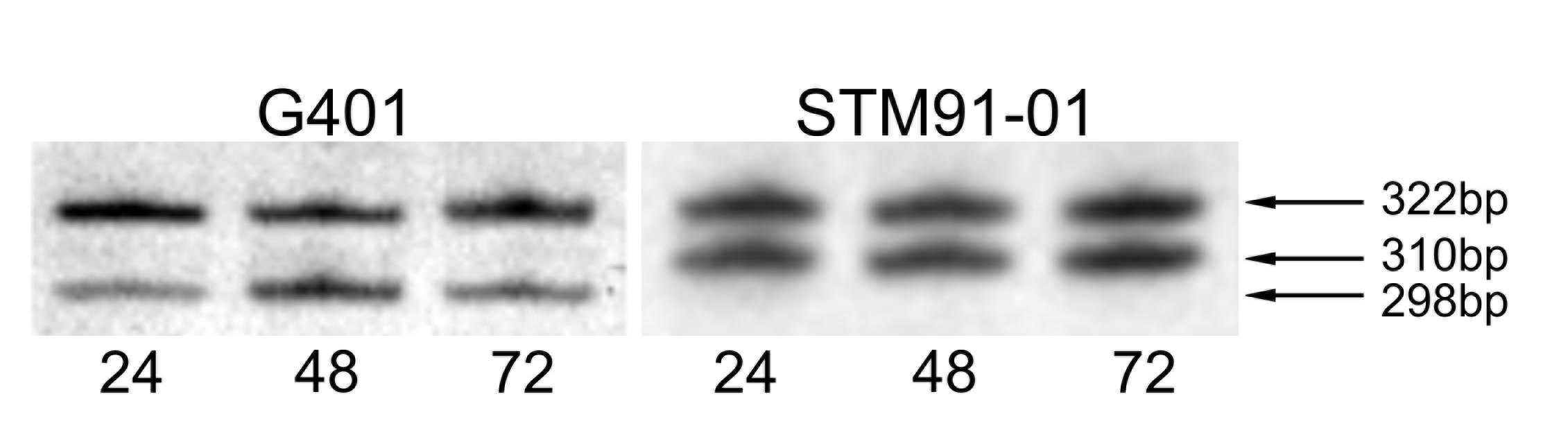

Supplement: Figure S3 — CDKN1C shows loss of imprinting in rhabdoid tumor. CDKN1C imprinting in G401 and STM91-01 rhabdoid tumor cell lines after 24, 48 and 72 hours in culture. CDKN1C alleles in G401 cells differed by 24 bp and those in STM91-01 differed by 12 bp. (0.08 MB TIF) [file pone.0004482.s003.tif]

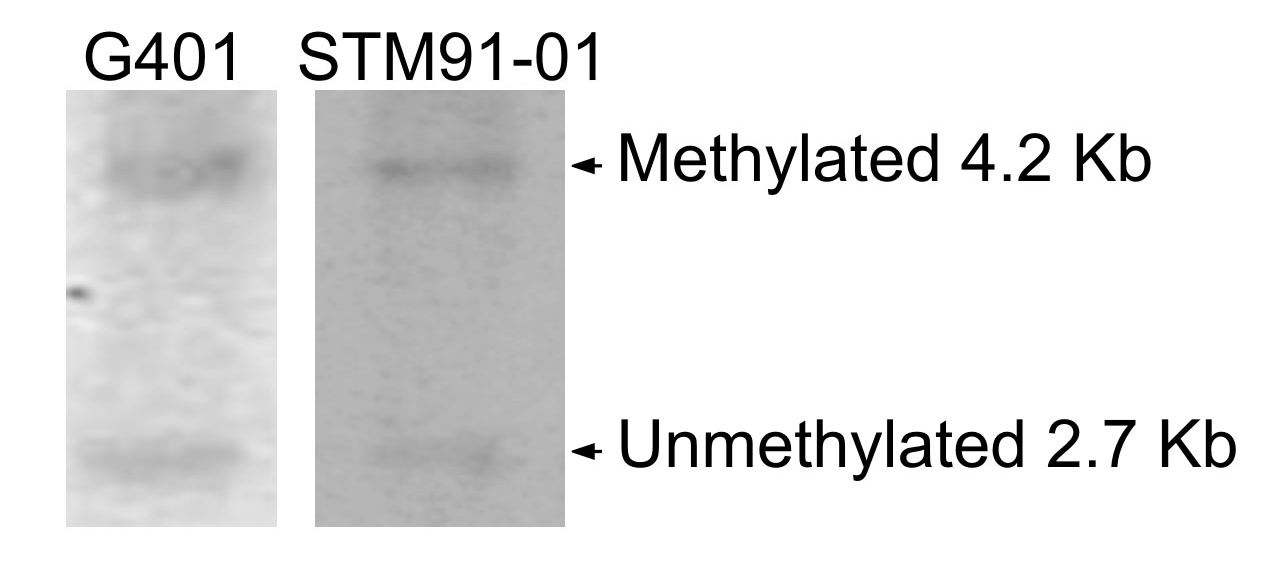

Supplement: Figure S4 — Normal LIT1 methylation is maintained in rhabdoid tumor. Methylation sensitive southern blotting at IC2 (LIT1) in G401 and STM91-01 cells showing normal patterns of methylation, with methylated (4.2 kb) and unmethylated (2.7 kb) bands of similar intensity. (0.06 MB TIF) [file pone.0004482.s004.tif]
