# Supplementary material for: Imprinted CDKN1C Is a Tumor Suppressor in Rhabdoid Tumor and Activated by Restoration of SMARCB1 and Histone Deacetylase Inhibitors
Source: PLoS One. 2009 Feb 16;4(2):e4482. doi: 10.1371/journal.pone.0004482 (PMC2637419; doi:10.1371/journal.pone.0004482)
Supplement: Table S1 — Romidepsin reduces proliferation in rhabdoid tumor cells. Table showing the percentage of cells in each cell cycle phase after 72 hours treatment with 1 nM Romidepsin and in controls containing 0.01% DMSO. The G401 data represent the mean values from three independent experiments and the STM91-01 data represent the mean values from six independent experiments. The error represents the standard error of the mean. (0.03 MB DOC) [file pone.0004482.s006.doc]

**Table S1**

| Cell cycle phase | % cells DMSO control | % cells 1nM Romidepsin |
| --- | --- | --- |
| G401 |  |  |
| G_0_ | 4.1 +/- 0.35 | 19.3 +/- 1.6 |
| G_1_ | 79.3 +/- 4.0 | 68.3 +/- 0.8 |
| G_2_SM | 16.3 +/- 3.6 | 11.5 +/- 1.85 |
|  |  |  |
| STM91-01 |  |  |
| G_0_ | 9.26 +/- 1.9 | 16.7 +/- 2.0 |
| G_1_ | 70.2 +/- 3.8 | 70.2 +/- 4.4 |
| G_2_SM | 20.8 +/- 4.0 | 12.7 +/- 3.6 |
